# Supplementary material for: Differential assembly of mouse and human tumor microenvironments
Source: Nat Immunol. 2026 May 19;27(6):1282–93. doi: 10.1038/s41590-026-02505-7 (PMC13226090; doi:10.1038/s41590-026-02505-7)
Supplement: Supplementary file 1 — Reporting Summary [file 41590_2026_2505_MOESM1_ESM.pdf]

nature portfolio

Corresponding author(s): Tristan Courau, Matthew Krummel

Last updated by author(s): Mar 10, 2026

Reporting Summary

Nature Portfolio wishes to improve the reproducibility of the work that we publish. This form provides structure for consistency and transparency in reporting. For further information on Nature Portfolio policies, see our Editorial Policies and the Editorial Policy Checklist.

Statistics

For all statistical analyses, confirm that the following items are present in the figure legend, table legend, main text, or Methods section.

n/a	Confirmed	
☐	☒	The exact sample size (*n*) for each experimental group/condition, given as a discrete number and unit of measurement
☐	☒	A statement on whether measurements were taken from distinct samples or whether the same sample was measured repeatedly
☐	☒	The statistical test(s) used AND whether they are one- or two-sided<br>*Only common tests should be described solely by name; describe more complex techniques in the Methods section.*
☒	☐	A description of all covariates tested
☐	☒	A description of any assumptions or corrections, such as tests of normality and adjustment for multiple comparisons
☐	☒	A full description of the statistical parameters including central tendency (e.g. means) or other basic estimates (e.g. regression coefficient) AND variation (e.g. standard deviation) or associated estimates of uncertainty (e.g. confidence intervals)
☐	☒	For null hypothesis testing, the test statistic (e.g. *F*, *t*, *r*) with confidence intervals, effect sizes, degrees of freedom and *P* value noted<br>*Give P values as exact values whenever suitable.*
☒	☐	For Bayesian analysis, information on the choice of priors and Markov chain Monte Carlo settings
☒	☐	For hierarchical and complex designs, identification of the appropriate level for tests and full reporting of outcomes
☐	☒	Estimates of effect sizes (e.g. Cohen's *d*, Pearson's *r*), indicating how they were calculated

Our web collection on statistics for biologists contains articles on many of the points above.

Software and code

Policy information about availability of computer code

Data collection	BD LSR Fortessa X20 (BD Biosciences), LSRFortessa (BD Biosciences), CyTOF 2 mass cytometer (Fluidigm), NovaSeq (Illumina) were used for data collection
Data analysis	Data analysis tools are described in the methods section of our manuscript. Flow cytometry data was collected using BD FACSDiva software and analyzed with FlowJo (BD). CyTOF data was collected using a CyTOF 2 Fluidigm mass cytometer, debarcoded and normalized in R using Premessa before analysis using FlowJo (BD). BulkRNAseq of human samples was described in Combes et al. Cell 2022. Single-cell analysis of human samples was described in Ray et al. Sci Immunol 2025. Single-cell analysis of mouse samples was performed using cellranger, Seurat and ggplot2 in R.

For manuscripts utilizing custom algorithms or software that are central to the research but not yet described in published literature, software must be made available to editors and reviewers. We strongly encourage code deposition in a community repository (e.g. GitHub). See the Nature Portfolio guidelines for submitting code & software for further information.

Data

Policy information about availability of data

All manuscripts must include a data availability statement. This statement should provide the following information, where applicable:

- Accession codes, unique identifiers, or web links for publicly available datasets
- A description of any restrictions on data availability
- For clinical datasets or third party data, please ensure that the statement adheres to our policy

Both human (https://quipi.org/app/quipi) and murine (https://quipi.org/app/quipi_humu) datasets can be readily queried and visualized using our user online interface. The Human raw data can be accessed as described in Combes et al. Cell 2022. The murine scRNAseq data has been publicly deposited in GEO under accession number GSE310560.

Research involving human participants, their data, or biological material

Policy information about studies with human participants or human data. See also policy information about sex, gender (identity/presentation), and sexual orientation and race, ethnicity and racism.

Reporting on sex and gender	Patient metadata recorded (described in Combes et al, Cell 2022) but not used to make comparisons based on sex and gender
Reporting on race, ethnicity, or other socially relevant groupings	Patient metadata recorded (described in Combes et al, Cell 2022) but not used to make comparisons based on race, ethnicity or other socially relevant groupings
Population characteristics	Patient metadata recorded and described in Combes et al, Cell 2022
Recruitment	Described in Combes et al, Cell 2022
Ethics oversight	All human tumor samples were collected with patient consent after surgical resection under a UCSF IRB approved protocol (UCSF IRB# 20-31740), under the UCSF ImmunoProfiler project as described in Combes et al, Cell 2022.

Note that full information on the approval of the study protocol must also be provided in the manuscript.

Field-specific reporting

Please select the one below that is the best fit for your research. If you are not sure, read the appropriate sections before making your selection.

☒ Life sciences ☐ Behavioural & social sciences ☐ Ecological, evolutionary & environmental sciences

For a reference copy of the document with all sections, see nature.com/documents/nr-reporting-summary-flat.pdf

Life sciences study design

All studies must disclose on these points even when the disclosure is negative.

Sample size	We used 2-14 mice per experimental group for all studies. A precise list of animal numbers per group per analysis is provided as a supplementary table with the manuscript. No statistical methods were used to pre-determine sample sizes.
Data exclusions	No data were excluded.
Replication	All mass cytometry and scRNAseq experiments were performed twice with multiple biological independent samples. Data was pooled as indicated in the relevant supplementary table.
Randomization	No randomization was needed in this study
Blinding	No blinding was done for this study

Reporting for specific materials, systems and methods

We require information from authors about some types of materials, experimental systems and methods used in many studies. Here, indicate whether each material, system or method listed is relevant to your study. If you are not sure if a list item applies to your research, read the appropriate section before selecting a response.

Materials & experimental systems

n/a	Involved in the study	
☐	☒	Antibodies
☐	☒	Eukaryotic cell lines
☒	☐	Palaeontology and archaeology
☐	☒	Animals and other organisms
☒	☐	Clinical data
☒	☐	Dual use research of concern
☒	☐	Plants

Methods

n/a	Involved in the study	
☒	☐	ChIP-seq
☐	☒	Flow cytometry
☒	☐	MRI-based neuroimaging

Antibodies

Antibodies used

Mouse CyTOF antibodies:
Specificity Supplier Reference Clone
B220 Biolegend 103202 RA3-6B2
CCR7 Biolegend 120101 4B12
CD103 Biolegend 121402 2E7
CD11b Biolegend 101202 M1/70
CD11c Biolegend 117302 N418
CD16/32 BD 553142 2.4G2
CD206 Biolegend 141702 C068C2
CD24 Biolegend 101802 M1/69
CD3e Biolegend 100202 17A2
CD301b Biolegend 146802 URA-1
CD38 Biolegend 102702 90
CD4 Biolegend 100506 RM4-5
CD44 Biolegend 103002 IM7
CD45 Biolegend 103102 30-F11
CD49b Biolegend 103513 HMa2
CD62L R&D MAB5761 MAB5761
CD64 Biolegend 139302 X54-5/7.1
CD69 R&D AF2386 Polyclonal
CD8 Biolegend 100702 53-6.7
CD86 Biolegend 105002 GL-1
CD90 Biolegend 105202 G7
cKit Biolegend 105802 2B8
CTLA-4 Biolegend 106302 UC10-4B9
F4/80 Biolegend 123102 BM8
FcER1a Biolegend 134302 MAR-1
Flt3 eBiosciences 14-1351-85 A2F10
Foxp3 eBiosciences 14-4771-80 NRRF-30
GATA3 Biolegend 653802 16E10A23
ICOS Biolegend 313502 C398.4A
Ki67 eBiosciences 14-5698-82 SolA15
Ly6C Biolegend 128002 HK1.4
Ly6G Biolegend 127602 1A8
MHC II Biolegend 107602 M5/114.15.2
PD-1 Biolegend 135202 29F.1A12
PD-L1 Biolegend 124302 10F.9G2
PDCA-1 Biolegend 127002 927
RORgt eBiosciences 14-6981-82 B2D
Siglec-F BD 552125 E50-2440
Siglec-H Biolegend 129602 551
SIRPa Biolegend 144002 P84
T-bet Biolegend 644802 4B10
TCRgd Biolegend 118101 GL3
Ter119 Biolegend 116202 Ter119
Tim-3 Biolegend 134002 B8.2C12

Mouse scRNAseq sorting panel:
Specificity Fluorochrome Supplier Clone Reference
B220 Alexa Fluor 488 Biolegend RA3-6B2 103225
CD49b PE Biolegend HMα2 103506
I-A/I-E Brilliant Violet 421 Biolegend M5/114.15.2 107632
CD11b Brilliant Violet 510 Biolegend M1/70 101245
CD11c Brilliant Violet 650 Biolegend N418 117339
CD90.2 Brilliant Violet 785 Biolegend 30-H12 105331
CD45.2 Brilliant Ultra Violet 395 BD 104 564616

Validation

All antibodies conjugated to fluorophores are commercially available and validated both by the manufacturer and through citations

Validation	in the scientific literature. Validation materials for each antibody are accessible on the respective manufacturer's homepage.<br><br>For mass cytometry, antibodies were purchased unlabeled and conjugated to heavy metals in-house. Antibody conjugation to heavy metal tags was done using the MaxPar Antibody Conjugation Kit (Fluidigm) according to the manufacturer's protocol. After labeling, antibodies were diluted to 0.2mg-0.5mg/mL in antibody stabilization solution (Candor Bioscience) and stored at 4°C until use. Before using experimentally, conjugated antibodies were titrated on mouse tissue to determine optimal staining concentration.

Eukaryotic cell lines

Policy information about cell lines and Sex and Gender in Research

Cell line source(s)	Cell line Origin Culture media Incubation Injection site # injected<br>B16-F10 ATCC CRL-6475 DMEM (Gibco 11995-065), 10% FCS (Benchmark), 1X Penicillin-Streptomycin-Glutamine (ThermoFisher) 37ºC 5% CO2 Subcutaneous, right flank 250K<br>LLC ATCC CRL-1642 37ºC 5% CO2 Subcutaneous, right flank 500K<br>MC38 Sigma-Aldrich SCC172 37ºC 5% CO2 Subcutaneous, right flank 500K<br>4T1 ATCC CRL-2539 37ºC 5% CO2 Mammary fat pad 250K<br>YUMM1.G1 ATCC CRL-3363 37ºC 5% CO2 Subcutaneous, right flank 2M<br>YUMM3.3 ATCC CRL-3365 37ºC 5% CO2 Subcutaneous, right flank 2M<br>YUMM5.2 ATCC CRL-3367 37ºC 5% CO2 Subcutaneous, right flank 2M<br>KPC-F (FC1245) Eric Collisson<br>(UCSF) 37ºC 5% CO2 Pancreas (orthotopic injections described in Jiang et al., Gastroenterology 2022) 1K<br>KPCY-C2 (6694c2) 37ºC 5% CO2 500K<br>KPCY-C5 (7160c5) 37ºC 5% CO2 500K<br>ID8 Fisher<br>Scientific SCC145 DMEM, 4% FCS, 1X Penicillin-Streptomycin-Glutamine, 1X Insulin-Transferrin-Selenium (ThermoFisher) 37ºC 5% CO2 Subcutaneous, right flank 2M<br>RENCA ATCC CRL-2947 RPMI (Gibco 11875-093), 10% FCS, 1X Penicillin-Streptomycin-Glutamine 37ºC 5% CO2 Subcutaneous, right flank 500K<br>CT26 ATCC CRL-2638 37ºC 5% CO2 Subcutaneous, right flank 500K
Authentication	No authentication was done in this study.
Mycoplasma contamination	All cell lines tested negative for mycoplasma.
Commonly misidentified lines<br>(See ICLAC register)	This study did not use misidentified lines.

Animals and other research organisms

Policy information about studies involving animals; ARRIVE guidelines recommended for reporting animal research, and Sex and Gender in Research

Laboratory animals	Besides MMT-PyMT mice, this study used 7-9 week old female C57BL6 from JAX, stock No. 000664.
Wild animals	No wild animals were used in this study.
Reporting on sex	Except when indicated, experiments were performed using female mice. No different outcomes were observed based on sex differences.
Field-collected samples	This study did not involve field-sample collections.
Ethics oversight	All procedures were approved by the Institutional Animal Care and User Committee (IACUC).

Note that full information on the approval of the study protocol must also be provided in the manuscript.

Plants

Seed stocks	N/A
Novel plant genotypes	N/A
Authentication	N/A

Flow Cytometry

Plots

Confirm that:

☒ The axis labels state the marker and fluorochrome used (e.g. CD4-FITC).

☒ The axis scales are clearly visible. Include numbers along axes only for bottom left plot of group (a 'group' is an analysis of identical markers).

☒ All plots are contour plots with outliers or pseudocolor plots.

☒ A numerical value for number of cells or percentage (with statistics) is provided.

Methodology

Sample preparation	CyTOF samples preparation:<br>After harvest, tumors were placed in a 12-well plate and minced to sub-millimeter pieces in 2mL of RPMI containing Collagenase IV (4 mg/mL) and DNase I (0.1mg/mL). Tissues were then incubated at 37C for 30 min, with a mechanical dissociation step using thorough pipetting after the first 15 minutes. Digestion was stopped by adding 2mL of cold RPMI + 10% FCS + 1X PS-Glu to each sample before filtering through a 100 mm mesh and centrifugation at 500 g for 5 min at 4C. Cells were then resuspended in RPMI + 10% FCS + 1X PS-Glu for further counting and analysis.<br><br>Mass cytometry (CyTOF) was performed as described elsewhere76. Briefly, conjugations of mass cytometry antibodies with metal isotopes were done using the Maxpar® conjugation kit (Fluidigm) according to manufacturer's protocols and each antibody was titrated to define its optimal staining concentration. Each freshly digested sample was first stained with cisplatinium, fixed in 3.2% PFA and frozen at -80C. For CyTOF staining, the samples were then thawed and barcoded by mass-tag labelling with distinct combinations of stable Pd isotopes in 0.02% saponin in PBS before further pooling and staining. For this, cells were first resuspended in cell-staining media (Fluidigm) containing metal-labeled antibodies against CD16/32 for 5 min at room temperature to block Fc receptors, followed by the addition of a cocktail containing surface markers antibodies in a final volume of 500µL for 30 min at room temperature. Cells were then permeabilized with methanol for 10 min at 4 °C, washed and incubated with a cocktail containing intracellular markers antibodies in a final volume of 500µL for 30 min at room temperature (all antibodies listed in Table S2). Cells were finally stained with 191/193Ir DNA intercalator (Fluidigm) diluted in PBS with 1.6% PFA 48h prior to data acquisition. For acquisition, cells were washed and resuspended at 1M/mL in deionized water + 10% EQ four element calibration beads (Fluidigm) and analyzed on a CyTOF mass cytometer (Fluidigm).<br><br>scRNAseq samples preparation:<br>For most mouse experiment, we started by sampling 1e6 cells from the tumor of each animal and generated a single pool for each group. A group of 5 mice therefore generated a pool of 5e6 cells. This cell pool was then stained with the Zombie NIR viability dye (1/1000 in PBS, 10min at 4C), before being incubated with Fc block (clone 2.4G2, Tonbo Biosciences) and barcoded with HTO antibodies (TotalSeq-A from BioLegend). We then pooled all barcoded samples together and stained them with mix of fluorescent-labelled antibodies (Table S2). Using a BD FACSAria II cell sorter (BD Biosciences), we then gated live immune cells (Zombie-CD45+) and sorted 2 pools of cells from these samples: a pool of lymphoid cells containing equal amounts of T cells (CD90.2+), B cells (B220+MHCII+) and NK cells (CD49b+), and another pool of myeloid cells gated as CD11b + and/or CD11c+ among the non-T-B-NK cells. These two pools were then washed, counted and then individually encapsulated following 10X Genomics specifications for v.3 3' chemistry.
Instrument	BD LSR Fortessa X20 (BD Biosciences), LSRFortessa (BD Biosciences), CyTOF 2 mass cytometer (Fluidigm)
Software	BD FACSDiva, FlowJo
Cell population abundance	Abundances of relevant cell populations are described across the manuscript.
Gating strategy	Gating strategy is provided in supplementary figures.

☒ Tick this box to confirm that a figure exemplifying the gating strategy is provided in the Supplementary Information.
